# The Role of Rehabilitation in the Treatment of Constipation in Oncological Patients

**DOI:** 10.3390/jcm12155083

**Published:** 2023-08-02

**Authors:** Rita Chiaramonte, Marco Bonfiglio, Salvatore Caramma, Rosaria Condorelli

**Affiliations:** 1 Department of Biomedical and Biotechnological Sciences, University of Catania, 95123 Catania, Italy; 2 Provincial Health Department of Siracusa, 96014 Sicily, Italy; 3 Department of Pain Management, Policlinico-San Marco Hospital, 95123 Catania, Italy; salvacaramma@tiscali.it; 4 Department of Medical Oncology, EOC—Istituto Oncologico della Svizzera Italiana, 6500 Bellinzona, Switzerland; rosaria.condorelli@eoc.ch

**Keywords:** neoplasms, constipation, rehabilitation, osteopathy

## Abstract

Background: Chemotherapy, as well as opioid and antiemetic drugs, can contribute to constipation in oncological patients. This systematic review aims to analyse the potential of specific rehabilitation strategies and alternative strategies for improving constipation symptoms, with the goal of incorporating these strategies into a dedicated protocol for managing cancer-related constipation. This could potentially reduce the dosages of or eliminate the need for constipation medications. Methods: A systematic search was conducted on PubMed, Scopus and Web of Science. The review included studies analysing constipation complaints in cancer patients treated with rehabilitation, acupuncture and osteopathy. Results: The review included 16 studies in line with PRISMA and PICOS criteria. Most studies showed that physical exercise, abdominal massage, TENS, acupuncture and education on the correct defecation position positively impacted the management of constipation and quality of life in oncological patients. A physiotherapy program involving massages as well as aerobic and resistance training improved constipation in oncological women, regardless of age, sex and frailty. A combination of abdominal massage, abdominal muscle stretching and education on proper defecation position alleviated the severity of constipation and related depression. However, the outcomes regarding TENS were yet inconsistent. Another technique, becoming increasingly common for constipation, and demonstrating positive results, involved stimulating trigger points through acupressure and acupuncture. Conversely, osteopathic and superficial manipulations more frequently required constipation medications than did the other alternative approaches. However, no existing studies have proposed a specific protocol to manage cancer-related constipation. Conclusions: The results of the studies confirm the positive influences of rehabilitation, osteopathy and acupuncture on constipation and pain in oncological patients. Nevertheless, further studies are required to establish the best type, timing and duration of treatment, as well as how the stage and location of the cancer and the cause of constipation (drug-induced or functional) impact the results.

## 1. Introduction

Constipation is one of the most common side effects of cancer therapy [1], negatively impacting daily living, well-being and quality of life. Furthermore, it is challenging to treat effectively. Specific drugs for constipation are not without adverse effects, necessitating additional treatment to manage this symptom.

According to the Rome Foundation criteria, functional and opioid-induced constipation is characterized by at least two of the following symptoms during 25% or more of defecations: straining, lumpy or hard stools, sensation of incomplete evacuation, sensation of anorectal obstruction or blockage, reliance on manual manoeuvres to promote defecation, or fewer than three unassisted bowel movements per week [2]. In palliative care services, it is estimated that patients who are most affected by cancer suffer primarily from pain in 64% of cases, anorexia in 34%, weakness in 32% and constipation in 32% [3]. Despite the high incidence rate, a unified strategy for treating symptoms related to constipation in cancer patients has yet to be proposed.

The causes of constipation can be divided into in primary (or functional) and secondary categories. Functional constipation is characterized by slow transit or outlet obstruction, and can be exacerbated by a low fibre diet, immobility, abdominal or pelvic surgery, and medications. Secondary causes include medications such as analgesics (especially opioids), antihypertensives like calcium channel blockers or diuretics, iron supplementation [4], and antiemetic drugs, including serotonin antagonists and anticholinergic drugs [5]. Additionally, chemotherapy can induce both constipation and diarrhoea, with medications such as alkylating agents (cisplatin and oxaliplatin), antimetabolites (gemcitabine), immunomodulating agents (thalidomide), and mitotic inhibitors (vincristine) [6].

The most common drug-related cause of constipation in oncological patients is opioid-induced constipation, due to the need for opioid usage in managing cancer pain. In fact, the incidence of chronic pain in cancer patients ranges from 13% to 91% [7,8], negatively impacting quality of life, increasing healthcare services usage and reducing work productivity [9]. Additionally, chemotherapy-induced constipation can significantly diminish a patient’s normal quality of life and lead to severe psychological symptoms, including anxiety and stress [10]. Moreover, the use of anti-serotonin drugs, such as the 5-hydroxytryptamine receptor antagonist, which are commonly included in cancer treatment regimens to alleviate emetogenesis, often exacerbates constipation severity [11].

Chemotherapy and opioid and antiemetic drugs can contribute to constipation in oncological patients. The usage of these drugs often necessitates additional interventions to alleviate constipation-related symptoms. However, to date, the existing literature has not offered any definitive and effective management protocols for this issue. The aim of this systematic review is to incorporate these strategies, such as acupuncture and osteopathy, that improve symptoms of constipation, and thus to include these strategies in a specific protocol for the management of cancer-related constipation, and reduce the dosages of or enable the avoidance of, constipation medications.

## 2. Materials and Methods

### 2.1. Protocol and Registration

This systematic review analysed the efficacy of various rehabilitation strategies and alternative approaches, such as osteopathy and acupuncture, for treating constipation in patients with cancer.

The review was registered in PROSPERO (CRD42023432357), and was conducted in accordance with the guidelines set out by the preferred reporting items for systematic reviews and metanalyses (PRISMA) [12], along with the population, intervention, comparison, outcome and study design (PICOS) criteria [13].

### 2.2. Eligibility Criteria

This systematic review adhered to the PRISMA and PICOS guidelines. It included studies that examined adults with cancer who were suffering from constipation (participants), managing the condition conservatively with rehabilitation programs and alternative approaches, either independently or in conjunction with anti-constipation drugs. Various forms of treatment were included, such as physical exercise, massage therapy, transcutaneous electrical nerve stimulation (TENS) and acupuncture (intervention). The comparators involved subjects who did not receive a specific rehabilitation treatment for constipation or who were given a conservative therapy other than drugs for constipation. The documented outcomes indicated differences in the quality of life and severity of constipation following treatments. The design of the included studies consisted of controlled studies with separate control groups.

In addition, any type of tumour, regardless of location, or stage (severity) was included.

Only original articles written in English were included. Studies whose samples comprised solely subjects not affected by cancer, those not experiencing constipation and those not treated with a rehabilitation program or other alternative approaches were excluded. Grey literature and unpublished data were not considered. Case studies and articles without a separate control group were also excluded.

### 2.3. Information Sources and Search Strategy

**Figure 1:**
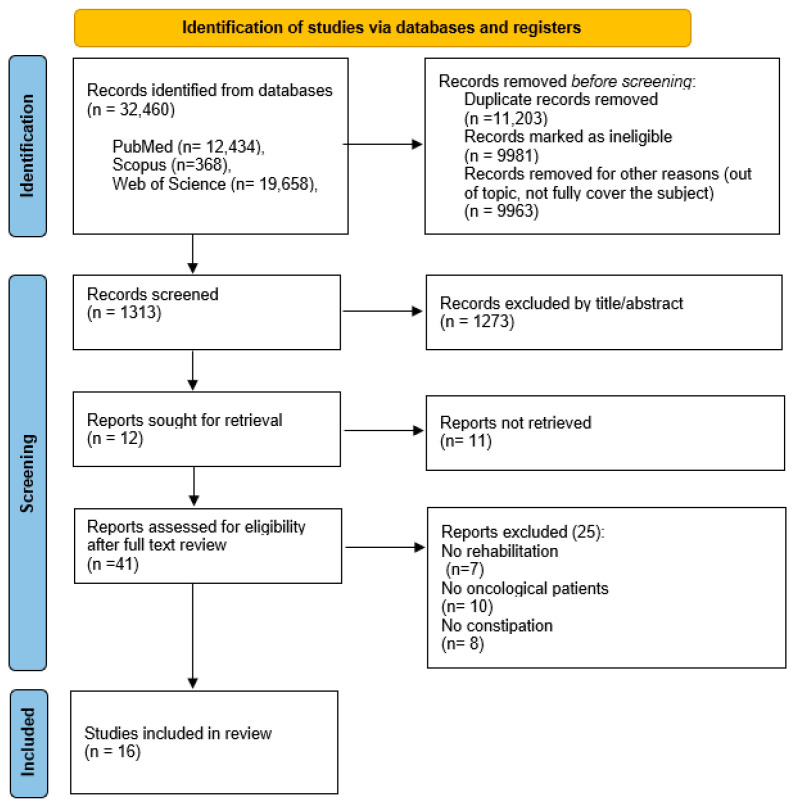
Flow-chart of the process of literature search and extraction of studies meeting the inclusion criteria.

The acquisition of relevant articles was accomplished using the search strategies described in Figure 1. The studies were grouped and organized according to the PICOS strategy [13], as illustrated in Table 1. The articles search was conducted in the databases PubMed, Scopus and Web of Science, up to June 2023. No restriction on publication dates (year of publication) were applied. The comprehensive search strategy was based on the Mesh keywords: “Rehabilitation” OR “Exercise” OR “Physical Therapy Modalities” AND “Constipation” OR “Opioid-Induced Constipation” AND “Neoplasm” OR “Carcinoma”. Other key searches and variations included: “Physical activity” OR “Training” OR “Physiotherapy” OR “Acupuncture” OR “Osteopathy” OR “Herbal medicine” AND “Stypsis” AND “Cancer” OR “Tumour” OR “Tumor” OR “Malignancy”.

The grading of recommendations, assessment, development and evaluation (GRADE) guidelines for systematic reviews were used to assess the quality of the results An evaluation of the quality of the outcomes was conducted to signify the degree [14,15,16,17,18]. of certainty (high, moderate, low, or very low) in the total effect estimates (Table 2).

**Table 1:** Characteristic of the included studies.

Authors Years	Study Design	Sample Size, Age, Location of Cancer, Treatment/Control Group	Groups	Causes of Constipation	Outcome measures	Rehabilitation, Osteopathy and Alternative Approaches	Timing Rehabilitation	Results	Conclusion
Cai 2019 [19]	RCT	251 pt 55.3 ± 3.1/ 51.2 ± 3.4 y - 48/41 lung - 9/6 pancreatic - 8/10 gastric - 24/30 breast - 3/2 cervical - 2/7 ovarian - 9/15 colon - 6/4 hepatic - 8/6 prostate - 4/6 melanoma - 4/4 metastatic Control group: - 4 lymphoma - 1 mesothelioma - 2 sarcoma	- Treatment group: 124 pt TENS + lactulose solution. - Control group 127 pt lactulose solution.	OIC	Bowel Function Index, PAC-QOL	TENS at bilateral ‘Tianshu’ and ‘Zusanli’ acupoints	15 mL of lactulose solution, twice per day for 14 days. TENS: once daily for 14 consecutive days, 30 min each time.	Defecation difficulty, incomplete defecation feeling and overall defecation satisfaction scores (*p* = 0.018).	TENS of relevant acupuncture points significantly relieved the symptoms of constipation in OIC and improved the quality of life.
Hanai 2016 [20]	RCT	30 breast cancer pt 55.3 ± 12.6/54.9 ± 9.7 y	- Intervention group. 15 pt. Start of the program before the first chemotherapy cycle. - Control group 15 pt. Start of the program on the day before their second chemotherapy.	Antiemetic-induced constipation	CAS, satisfaction of pt	Abdominal massage, abdominal muscle stretching, and education on proper defecation position.	1 min of massage/10 times per day	CAS *p* = 0.02, volume of stool *p* = 0.03, decrease in depression and dejection *p* = 0.02. Program satisfaction 43.6 and 26.4% as excellent and good.	The program’s results effective for mitigating the symptoms of antiemetic-induced constipation during chemotherapy
He 2021 [21]	Multi-center, RCT	171 pancreatic cancer pt 51.2 ± 10.7/58.9δ ± 9.9 y	- TENS group 87 pt. - Control group 84 pt.	Analgesic -induced constipation	NRS	TENS of the following points: on 1.5 cm away from middle line of T8 to T12 vertebra (belong to acupoints of B3, BL18, BL19, BL20, and BL21 in traditional Chinese meridian theory system), RN12, and pain point on abdomen.	30 min per day	NRS in TENS group has been largely decreased 77.9% after treatment and 27.1% in 2 h, before applying any analgesic medication. Pain was significantly well-controlled without analgesic medication supplement in TENS group (NRS *p* < 0.01) and 3 h after treatment (*p* < 0.01), and this analgesic effect lasted to 3 weeks after treatment cycle (NRS < 0.01).	With the treatment of TENS, 100% of patients with constipation had different degrees of improvement, and 91% gained better appetite.
Jensen 2014 [22]	RCT	107 Surgical urooncology pt after radical cystectomy with lymph node dissection. 69 (66–72)/71 (68–73) y	- Interventional group 50 pt. Pre and postoperative rehabilitation. - Standard group 57 pt. Standardized exercise program.	No specified constipation	HRQoL, EORTC QLQ-C30, satisfaction survey at discharge	Pre- and postoperative rehabilitation with mobilization and walking.	Scheduled time out of bed increasing from 3 h on day 1 after surgery to 8 h on the 4th post operative day	HRQoL scores in dyspnea (*p* < 0.05), constipation (*p* = 0.02), and abdominal flatulence (*p* < 0.05).	Pre- and postoperative rehabilitation can significantly and positively impact HRQoL aspects related to bowel management and respiratory function (dyspnea) without compromising inpatient satisfaction.
Lagrange 2019 [23]	RCT	94 breast cancer pt 57.2 ± 10.2/ 54.4 ± 12.1	- Visceral osteopathic manipulation 54 pt. - Control group superficial/soft tissue manipulation 40 pt.	Adjuvant chemotherapy related constipation	EORTC QLQ-C-30	Visceral osteopathy	During the first 3 cycles of chemotherapy of 15 min	No significant differences were found between the two groups concerning the rate of nausea/vomiting (*p* = 0.569) or constipation (*p* = 0.204). Patient reported impact of constipation and diarrhoea on quality of life was significantly lower in experimental group (*p* = 0.036 and *p* = 0.038, respectively).	Osteopathy does not reduce the incidence of nausea/vomiting in women operated on for breast cancer and undergoing adjuvant chemotherapy, but improves digestive quality of life.
Lai 2011 [24]	RCT	32 pt Age: 31–40 1 pt 41–50 5 pt 61–70 1 pt 71–80 11 pt 81–90 2 pt - 18 lung - 5 naso-pharyngeal - 2 breast - 1 uteri - 1 prostate - 1 pancreas - 4 unknown primary	- Aroma massage group 13 pt. - Plain massage group 11 pt. - Control group 8 pt.	Generical constipation	CAS, MQOL-HK	Aroma massage	Daily for 5 consecutive days, 15–20 min	Improved physical domain (*p* = 0.014). Significant decrease of the severity of constipation (*p* = 0.002).	Aroma massage helps to relieve constipation in patients with advanced cancer.
Lehmann 2023 [25]	Longitudinal Observational Study	4150 pt 33.1 ± 4.8 56.9 ± 7.0 76.2 ± 4.6 y - 285 head and neck - 75 esophageal - 149 gastric - 327 colon - 184 rectal - 38 liver - 118 pancreas - 273 lung - 50 skin - 1965 breast - 182 uterine - 200 ovarian - 441 prostate - 58 testicular - 115 renal - 122 bladder - 77 brain - 52 thyroid - 369 lymphoma - 88 myeloma - 108 leukaemia - 291 other	- Group A < 40 y. - Group B 40–70 y. - Group C > 70 y.	No specified constipation	EORTC QLQ-C-30, HADS, Norton score	Physiotherapy: massages, aerobic and resistance training.	The program comprised 21 days of rehabilitation with 2–3 h of therapeutic units per working day.	Significantly higher levels of social, emotional and cognitive functioning and a better global health/QOL (*p* < 0.05). Higher levels of pain, sleep disturbances, dyspnoea, and constipation (*p* < 0.05). Men reported higher appetite loss and diarrhoea (*p* < 0.001).	QOL is improved by rehabilitation in all patients’ groups, independently from age, sex, or the risk status.
Li 2020 [26]	RCT	150 pt with brain tumour 18–70 y	- Nontreatment group 50 pt. - Acupuncture group 50 pt. - Electro-acupuncture group 50 pt.	Post-operative constipation	Rome III diagnostic criteria, Cleveland Clinic constipation score, symptom assessment, PAC-QOL, self-rating anxiety scale, self-rating depression scale	Electro-acupuncture stimulation at Tiensu, 5- to 10-mA current, 15 Hz, and wave width 20 μs.	Once per day for 7 days consecutively.	Efficacy of electro-acupuncture *p* < 0.01. PAC-QOL not statistically significant (*p* > 0.05).	Acupuncture and electroacupuncture were effective in relieving postoperative constipation. Electroacupuncture decreased constipation, but it did not improve quality of life.
Nakano 2019 [27]	RCT	Advanced cancer 20 pt 70.0 ± 6.3 y - 5 Esophagus - 3 head and neck - 3 breasts - 2 kidneys - 2 lymphoma - 1 prostate - 1 liver - 1 lung - 2 other	- TENS group 10 pt. - No TENS group 10 pt.	No specified constipation	EORTC QLQ-C15-PAL	TENS on both ankle joints (10 Hz).	For 5 days.	No significative improvement in constipation (*p* = 0.75).	TENS reduced pain and the opioid rescue doses; it tended to improve nausea and appetite loss, but not constipation.
Shin 2016 [28]	RCT	Breast cancer 52 pt - 30–39 y 5/3 - 40–49 10/8 ≥50 11/15 pt	- Auricular acupressure group: 26 pt. - Control group 26 pt. (usual care)	Chemotherapy-induced constipation	CAS, Stool-form score, PAC-QOL	Auricular acupressure at 7 acupoints (intestine, rectum, San Jiao, spleen, lung, sympathetic, subcortex).	7 auricular acupoints, once per week for 6 consecutive weeks using vaccaria seeds.	CAS *p* < 0.001, Stool-form scores *p* = 0.003, and PAC-QOL *p* < 0.001.	Auricular acupressure improved stool form and quality of life and reduced CAS.
Song 2020 [29]	RCT	Malignant tumor 126 pt - Group A Acupoints 62.53 ± 11.89 y - Group B Chinese medication 61.88 ± 9.43 y - Group C Western medication 60.53 ± 10.89 y	- Acupoint group 41 pt. - Chinese herbal medication group 42 pt. - Western medication group with polyethylene glycol electrolyte powder 43 pt.	OIC	Constipation symptom score, PAC-QOL, serun nitric oxide synthetase (NOS)	Acupuncture at Huángqí, Ròucōngróng, Báizhú, Hòupò, Zhĭshí, Bīngláng, Láifúzĭ, Huŏmárén, Qīngpí. Herbal drugs were soaked in 1000 mL clear water for half an hour and decocted and condensed to 4 mL.	Once per day, 30 min each time for 14 days in total.	Constipation symptoms in the 3 groups, (*p* < 0.01). The improvements in acupoint group *p* < 0.05, PAC-QOL *p* < 0.05.	The ultrasonic penetration of herbal formula at Tiānshū (天枢 ST25) is effective on OIC.
Wang 2019 [30]	Non-R CT	30 pt with advanced cancer. 30–49 y 8 pt, 50–69 y 28 pt, 70–89 y 24 pt - intervention group acupuncture - Control group routine medical care	- Electro-acupuncture group 15 pt. - Sham electro-acupuncture group 15 pt.	Constipation in advanced cancer	Bristol stool form scale, four-point Likert scale for symptoms, VAS for discomfort	Acupressure at Zhongwan, Guanyuan, Tianshu.	8 min acupressure treatment for 3 consecutive days.	Symptom of constipation *p* < 0.001, hard stools *p* = 0.002; sensation of incomplete evacuation *p* < 0.001; sensation of anorectal obstruction *p* = 0.002, Bristol stool *p* < 0.001, comfort levels during defecation *p* < 0.001, colonic motility *p* < 0.001.	Improvement in symptoms of constipation, as motility, straining, hard stools, sensation of incomplete evacuation, and anorectal obstruction.
Wang 2022 [31]	RCT	100 cancer pt 18–85 y - 30–49 y 4 pt - 50–69 y 14 pt - 70–89 y 12 pt - 17/21 lung - 6/4 liver - 5/3 breast - 4/2 pancreas - 2/3 uterine - 3/1 nasopharynx - 2/1 colorectal - 2/1 stomach - 9/14 other	- Electro-acupuncture group 50 pt. - Sham group 50 pt.	OIC	Bristol stool form scale, number of spontaneous bowel movements/week, PAC-QOL, patients’ global assessment of treatment effectiveness, patients’ expectations.	Electro-acupuncture at bilateral Tianshu, Fujie, Shangjuxu for 30 min, with a continuous wave of 10 Hz and current intensity of 0.5 to 4 mA.	24-session treatment over 8 weeks, three sessions per week and 30 min each session. Follow up for 8 weeks.	Stronger evidence as to whether electro-acupuncture is an effective treatment for OIC among cancer patients.	Electroacupuncture was effective and safe for the treatment of OIC among patients with cancer.
Wang 2023 [32]	RCT	100 cancer pt 63.6 ± 10.4/65.1 ± 10.6 y - 17/21 lung - 6/4 liver - 5/3 breast - 4/2 pancreas - 2/3 uterine - 3/1 nasopharynx - 2/1 colorectal - 2/1 stomach - 9/14 other	- Electro-acupuncture group 50 pt. - Sham group 50 pt.	OIC	Bristol stool form scale, number of spontaneous bowel movements/week, PAC-QOL, patients’ expectations.	Electro-acupuncture at bilateral Tianshu, Fujie, Shangjuxu, with a continuous wave of 10 Hz and a current intensity of 0.5 to 4 mA.	24- session treatment over 8 weeks, three sessions per week and 30 min each session. Follow up for 8 weeks.	The proportion of overall responders at week 8 was 40.1% in the electro-acupuncture group and 9.0% in the sham group (*p* < 0.001).	Electroacupuncture increases spontaneous bowel movements with a good level of safety and quality of life.
Yıldırım 2019 [33]	RCT	Experimental l204 pt 60.5 ± 14.5/61.1 ± 13.2 y - 34/28 lung - 13/14 genito-urinary - 4/6 breast - 5/1 bone - 3/7 hematologic - 6/1 sarcoma - 2/4 head and Neck - 35/37 no cancer pt	- Video-guided abdominal massage 103 pt. - Control group (clinic approach) 102 pt.	OIC	Defecation diary, vas, PAC-QOL, Bristol stool scale	- Experimental group self-abdominal massage according to a video-guided abdominal massage. - Control group: clinic approach.	15-min of abdominal massage, for 4 weeks, twice per day, 30 min after breakfast and dinner.	Severity of constipation, straining, anal pain and bloating (*p* < 0.05), feeling of incomplete bowel emptying, better stool consistency (*p* < 0.05), increased number of defecations and quality of life scores *p* < 0.05.	The abdominal massage increased the number of defecations by 13% and it was an effective approach for managing OIC.
Yildirim 2022 [34]	RCT	140 pt - 60.9 ± 14.05/60.2 ± 12.8 y - 39/35 lung - 13/14 genito-urinary - 4/6 breast - 5/5 bone - 3/7 hematologic - 6/3 sarcoma	- Acupressure group A: 70 pt. - Control group: 70 pt. (routine treatment)	OIC	Defecation Diary, VASQ, PAC-QOL	Acupressure at Zhongwan, Guanyuan, Tianshu, acupoints.	8-min once per day for 4 weeks.	Stool consistency *p* = 0.001, straining *p* = 0.001, incomplete evacuation *p* = 0.001, stool amount *p* = 0.001, number of defecations *p* = 0.001.	A 4-week duration of acupressure was an effective way to improve the quality of life and to reduce the constipation symptoms in OIC.

Patients, pt; Years, y; Randomized Controlled Trial, RCT; Non-Randomized Controlled Trial, Non-RCT; Opioid-Induced Constipation, OIC; Transcutaneous Electrical Nerve Stimulation, TENS; Patient Assessment of Constipation Quality of Life Questionnaire, PAC-QOL; European Organisation for Research and Treatment of Cancer, EORTC; Quality of Life Questionnaire Quality of Core, QLQ-C; Visual Analogue Scale Questionnaire, VASQ; Constipation Assessment Scale, CAS; Numerous Rating Scale, NRS; Health-Related Quality of Life, HRQoL; McGill Quality of Life for Hong Kong Chinese, MQOL-HK; Hospital Anxiety and Depression Scale Questionnaires, HADS; Minutes, min.

**Table 2:** Summary of risk of bias and quality for each included study (original table) [17].

Study, Year	Random Sequence Generation	Allocation Concealment	Blinding Participants	Blinding of Outcome Assessment	Incomplete Data	Selective Reporting	Other Bias	Risk of Bias
Cai 2019 [19]	+	+	-	-	+	+	+	Low
Hanai 2016 [20]	+	+	-	-	+	+	+	Low
He 2021 [21]	+	+	+	+	+	+	+	Very low
Jensen 2014 [22]	+	+	+	+	+	+	+	Very low
Lagrange 2019 [23]	+	+	+	+	+	+	+	Very low
Lai 2011 [24]	+	+	-	-	+	+	+	Low
Li 2020 [26]	+	+	+	+	+	+	+	Very low
Nakano 2019 [27]	+	+	-	-	+	+	+	Low
Shin 2016 [28]	+	+	-	-	+	+	+	Very low
Song 2020 [29]	+	+	-	-	+	+	+	Low
Wang 2019 [30]	-	-	-	-	+	+	+	Moderate
Wang 2022 [31]	+	+	+	+	+	+	+	Very low
Wang 2023 [32]	+	+	+	+	+	+	+	Very low
Yıldırım 2019 [33]	+	+	-	-	+	+	+	Low
Yildirim 2022 [34]	+	+	+	+	+	+	+	Very low
**Study, Year**	**Bias Due to** **Confounding**	**Bias in Selection of Participants for the Study**	**Bias in** **Classification of Interventions**	**Bias Due to** **Deviations from** **Intended Intervention**	**Bias Due to Missing Data**	**Bias in** **M** **easurement of Outcomes**	**Bias in** **Selection of the Reported Result**	**Risk of Bias**
Lehmann 2023 [25]	+	+	+	+	+	+	+	Low
Quality assesssment					Summery of findings		Quality of evidence GRADE	
N. of studies	Limitations	Inconsistency	Indirectness	Pubblication bias	Sample characteristics		High	
16 studies	No significant limitations	No serious inconsistency	No serious indirectness	Unlikely	Population: oncological adults Intervention: rehabilitation (posture, physical exercises, massage, TENS), acupuncture, osteopathy Comparison: different therapeutic strategies, no treatment Outcomes: improvement of constipation symptoms and quality of life			

+ indicates reporting in full with low risk of bias; / indicates partial reporting with unclear risk of bias; - indicates no reporting with high risk of bias.

### 2.4. Study Selection

The selection of articles, along with the assessment of titles and abstracts, was conducted by two independent authors who were blinded to each other’s work, and who strictly adhered to the inclusion and exclusion criteria. After this preliminary screening, the remaining articles were critically evaluated to determine their eligibility for inclusion in the review. Additionally, the reference lists of the included studies were scanned for any further suitable studies that the initial search might have missed. In instances of disagreement between researchers regarding the selection of studies, a third author was consulted for resolution.

### 2.5. Assessment of Risk of Bias

The methodological quality of clinical trials was evaluated by two independent authors using the Cochrane risk of bias tool [35] and the risk of bias in non-randomised studies of interventions (ROBINS-I), in accordance with the Cochrane methodology [36,37]. The risk of bias in the selected clinical trials was categorized as high, moderate, low, very low, or unclear. The categories for risk of bias included random sequence generation, allocation concealment, and blinding of participants and personnel, as well as outcome assessment, incomplete outcome data and other sources of bias. Clinical trials selected were considered to have a moderate risk of bias if more than two of the criteria were rated as having a high or unclear risk (Table 2).

### 2.6. Data Synthesis

The systematic review identified a common theme among the studies: the roles of rehabilitation, osteopathy and other alternative approaches in managing constipation in cancer patients. A meta-analysis was not conducted due to the heterogeneous data about the type and timing of treatment, cause of constipation, location and stage of cancer and clinical condition of the patients.

## 3. Results

### 3.1. Study Selection

The search yielded 12,434 articles in PubMed, 368 in Scopus, and 19,658 in Web of Science, with the search focusing on clinical trials in English, and involving adult subjects (19+ years). After excluding duplicates, ineligible studies and those outside of the topic, 1313 articles were screened by title and abstract. Consequently, a total of 41 articles were assessed for eligibility following a full text review. Out of these, seven studies were excluded because they did not report any therapeutical plan, ten did not involve oncological patients, and eight did not pertain to constipation. Therefore, 16 publications were selected for inclusion.

### 3.2. Study Characteristics

Table 1 presents the 16 selected studies. Of these studies, 15 were clinical trials comparing rehabilitation, osteopathy intervention, or acupuncture with (a) the constipation medication alone [19,29,33], (b) no other treatment (control or sham group) [21,24,26,27,28,30,31,32,34], (c) different therapeutic programs (traditional versus pre- and postoperative rehabilitation program [22], different abdominal massage/manipulation [23,24] and acupuncture versus electroacupuncture [26]). Only one article reported a longitudinal observational study that compared different types of patients by age and sex, while also describing cancer localization [25]. Given its highly focused theme, the large sample size (6.757 patients), and the presence of control groups, it was included (Table 2) [25].

### 3.3. Rehabilitation, Osteopathy and Acupuncture

Although physical exercise, abdominal massage, TENS and acupuncture have been suggested for managing constipation in oncological patients, no existing studies have proposed a specific protocol for managing cancer-related constipation. A physiotherapy program consisting of massages and aerobic and resistance training was shown to improve constipation symptoms in women, regardless of age, sex and frailty status [25]. The combination of abdominal massage, abdominal muscle stretching and education on proper defecation posture helped to alleviate constipation severity and related depression [20]. Notably, the abdominal massage appeared to reduce constipation symptoms and improve quality of life [33] by enhancing stool consistency, reducing straining during defecation, mitigating the feeling of incomplete emptying after defecation, and increasing the number of defecations. Furthermore, the number of defecations increased by 13% [33]. Another study reported an improvement in quality of life related to digestive disorders due to visceral osteopathic manipulations. In contrast, patients who underwent superficial manipulation tended to require constipation medication more frequently [23].

The outcomes regarding TENS were not consistent. According to He et al. [21], 100% of patients experienced varying degrees of improvement through TENS, and 91% of patients with poor appetite noted better appetite. Moreover, TENS, especially when applied to specific sites, namely at bilateral ‘Tianshu’ (ST25 located at the middle of the abdomen, 2 cm lateral to the umbilicus) and ‘Zusanli’ (ST36 located on the tibialis anterior muscle four finger breadths below the kneecap and one finger breadth lateral from the anterior crest of the tibia) acupoints, seemed to decrease defecation difficulty and feelings of incomplete defecation, and improve defecation satisfaction [19]. Conversely, another study indicated that TENS on both ankle joints was not effective for constipation [27], but that it was effective for pain, with TENS placed on the back at the dermatomal level corresponding to the painful part or internal organs. Therefore, a technique could be suggested to reduce the dosage of opioid drugs, with a positive impact on opioid-induced constipation.

Another increasingly common technique for constipation involves the activation/stimulation of trigger points through acupressure and acupuncture. Acupressure appears to reduce pain, maintain patient relief, prevent nausea and constipation, and promote gastrointestinal motility and digestive juice secretion in oncological patients [29,30,34]. Similarly, electroacupuncture seems to have positive effects in facilitating rapid recovery of patients with constipation, both physically and psychologically [26,31,32]. As proposed for the application of TENS [19], the most commonly used acupuncture point was ‘Tianshu’ (ST25) [29,30,31,32,34]. Lastly, auricular acupressure also had a positive effect on constipation symptoms and improved quality of life [28].

There was a significant variability in the anti-constipation techniques proposed, particularly regarding the methods and timing of treatment. In terms of massage, recommendations included 1 min sessions performed 10 times per day on the abdomen [20], sessions performed twice daily for 4 weeks, 30 min after breakfast and dinner [33], or 5 days of sessions of 15–20 min each [24]. Visceral osteopathy was suggested after each cycle of chemotherapy (three sessions of 15 min each). For specific rehabilitation strategies, the progression from 3 h of mobilization and walking on the first day after surgery to 8 h on the fourth postoperative day was proposed [22]. Additionally, a 21-day program of physiotherapy featuring massages and aerobic and resistance training consisting of 2–3 h of therapeutic units per working day [25] was suggested for its anti-constipation effect. Moreover, the current literature proposes TENS on both ankle joints (10 Hz) for 5 days [27], or once daily for 14 consecutive days, 30 min time each, at acupuncture points (Tianshu’ and ‘Zusanli’) [19] 1.5 cm away from the midline of the T8 to T12 vertebrae (belonging to acupoints of B3, BL18, BL19, BL20 and BL21 in the traditional Chinese meridian theory system), RN12, and the pain point on the abdomen for 30 min [21]. However, electro-acupuncture stimulation was suggested at the Tiensu point, once per day for 7 consecutive days [26], or at the bilateral Tianshu, Fujie, Shangjuxu points for 30 min across a 24-session treatment over 8 weeks [31], or at the bilateral Tianshu, Fujie, Shangjuxu points, for 30 min each session across a 24-session treatment over 8 weeks, three sessions per week [32]. Acupuncture was suggested at Huángqí, Ròucōngróng, Báizhú, Hòupò, Zhĭshí, Bīngláng, Láifúzĭ, Huŏmárén and Qīngpí, once per day for a total of 14 days [29]. Lastly, auricular acupressure was suggested at seven acupoints (intestine, rectum, San Jiao, spleen, lung, sympathetic and subcortex) once per week for 6 consecutive weeks [28], or at the Zhongwan, Guanyuan and Tianshu acupoints for 8 min once per day for 3 consecutive days [30], or for 4 weeks [34].

### 3.4. Constipation Assessment

The assessment of constipation in oncological patients encompassed the evaluation of symptoms and quality of life. Symptoms were investigated through measures such as the number of spontaneous bowel movements per week [31,32], the Bristol stool form score [28,30,31,32,33], the constipation assessment scale (CAS) [20,24,28]. The Cleveland Clinic constipation score [26] was also used. Quality of life was examined through the Patient Assessment of Constipation Quality of Life Questionnaire (PAC-QOL) [19,26,28,29,31,32,33,34], the European Organisation for Research and Treatment of Cancer EORTC Quality of life Core Questionnaire (EORTC QLQ-C) [22,23,25,27], the health-related quality of life score (HRQoL) [22] and the McGill Quality of Life Questionnaire (MQOL) [24]. Moreover, mood was studied with generic scales such as the Hospital Anxiety and Depression Scale (HADS) Questionnaires [25], the self-rating anxiety scale, and the self-rating depression scale [26].

Despite the wide variety of strategies proposed, the current literature indicates that all treatments reduced the severity of constipation, according to the CAS through abdominal massage and auricular acupressure [20,24,28], and controlled symptoms, as reported by the defecation diary for abdominal massage and acupressure [33,34], the four-point Likert scale for electro-acupuncture [30], the Rome III Diagnostic Criteria, the Cleveland Clinic constipation score [26] and the constipation symptom score [29] for acupuncture. Moreover, electro-acupuncture increased the number of spontaneous bowel movements per week [31,32], softened the stool according to the Bristol stool form scale [30,31,32], and improved the stool-form score through auricular acupressure [28]. It also eased and reduced the feeling of incomplete bowel evacuation according to the bowel function index through TENS [19]. Furthermore, most studies showed an improvement in the quality of life according to the EORTC QLQ-C-30 after massages and aerobic and resistance training [22,25], HRQoL after mobilization and walking [22], MQOL-HK after massage [24], and PAC-QOL through TENS, auricular acupressure, acupuncture and electro-acupuncture, abdominal massage and acupressure [19,28,29,31,32,33,34]. Only a few articles documented a lack of difference in quality of life, specifically, according to EORTC QLQ-C-30 after visceral osteopathic manipulation [23], EORTC QLQ-C-15 after TENS on both ankle joints [27], and PAC-QOL after electro-acupuncture stimulation at the Tiensu point [26]. Likewise, the reduction of constipation seemed to correlate with reductions in anxiety and depressive mood, as recorded with HADS after massages and aerobic and resistance training [25], and the self-rating anxiety scale [26] and self-rating depression scale after electro-acupuncture stimulation at the Tiensu point [26]. Lastly, patients also reported satisfaction linked to the therapy according to the Patients’ Global Assessment of Treatment Effectiveness [31], Patients’ Expectations Through Electro-Acupuncture [31,32], and a satisfaction survey after mobilization and walking [22].

## 4. Discussion

Oncological patients have a high risk of developing constipation due to the side effects of chemotherapy, antiemetics and, particularly, opioid drugs. This side effect adds to the other, more well-known symptoms, such as fatigue, nausea, muscle pain and neuropathic pain.

Constipation impacts the quality of life, leading to psychological and social problems related to physical discomfort, mental distress and reminders of mortality, often resulting in avoidance and social isolation [38]. Therefore, this side effect generally necessitates additional pharmacologic and rehabilitative interventions, as well as alternative strategies, like acupuncture and osteopathy. Additionally, it is crucial to implement a shared protocol that encourages a healthy lifestyle, dietary strategies such as fibre supplements, and increased water intake.

According to our research, physical exercise, abdominal massage, TENS and acupuncture have been proven to be safe and effective in managing constipation in oncological patients. However, the areas of rehabilitation, osteopathy and acupuncture have not received sufficient attention. In fact, no existing studies have proposed a specific protocol for managing cancer-related constipation.

In developing a program that combines osteopathy, acupuncture and rehabilitation, the objectives should be aimed at reducing constipation symptoms, improving quality of life, and decreasing the reliance on constipation medications laden with side effects. To achieve these outcomes, all the suggested treatments can be considered and integrated with each other, always taking into account the patient’s clinical condition and fatigue levels, especially when it comes to aerobic and resistance training. With regard to this latter point, the efficacies of massages, aerobic exercises, and resistance training have been observed regardless of age, sex and frailty status [25]. However, the effectiveness of the training is significantly influenced by the patient’s overall well-being. Indeed, cancer-related fatigue could reduce adherence to the program and affect its outcomes.

Furthermore, while exercise is generally beneficial, current evidence suggests that bed rest does not pose a risk factor for constipation [39]. As such, its effects on constipation in cancer patients remain unclear and relatively under-studied in the literature.

According to the research, while all the described techniques have demonstrated positive effects on constipation, the outcomes from TENS are inconsistent, yielding positive results in some studies [19,21] and insignificant results in others [19,21,27]. This discrepancy could be due to the variability in application points, as this technique is operator-dependent.

Undoubtedly, another significant factor for oncological patients and caregivers is the provision of effective counselling. Indeed, proper advice on self-care management, which may include abdominal massage, abdominal muscle stretching and education on the appropriate defecation position, should be clearly explained and implemented on a daily basis [20].

Unfortunately, the cause of constipation is not always identified in the studies [22,24,25,27,30], nor is the stage of tumour identified in all the included research. However, most of the studies focus on the opioid-induced constipation [19,29,31,32,33,34] and analgesic-induced constipation [21]. A few studies tackle chemotherapy-related constipation [23,28]; one discusses antiemetic-induced constipation [20], and another, post-operative constipation [26]. This heterogeneity, combined with the lack of information about the tumour stage, makes it challenging to stratify the results in order to better comprehend how treatment outcomes may depend on the various causes of constipation.

High heterogeneity was also found for the sites of tumours. Indeed, the treatment of constipation in patients affected by breast cancer was the most studied, likely due to its high prevalence in younger women. In cases of breast cancer, methods such as massage, abdominal muscle stretching and education on proper defecation position were found to reduce antiemetic-induced constipation [20]. Additionally, visceral osteopathy [25] and auricular acupressure [28] were proposed for treating chemotherapy-related constipation, while aerobic and resistance training were recommended for unspecified types of constipation [25]. TENS of relevant acupuncture points [19], massage [33], electro-acupuncture [31,32] and acupressure [34] significantly alleviated symptoms of constipation in cases of opioid-induced constipation. Meanwhile, TENS of ankle was proposed for unspecified constipation [27].

Pancreatic, lung and genito-urinary involvement also seemed to benefits from TENS [19] and electro-acupuncture [31,32] in cases of opioid-induced constipation. Specifically, for pancreatic and lung cancer patients, massage [33] for opioid-induced constipation, TENS for analgesic-induced constipation [21] and TENS of the ankle [27], massage [24] and aerobic and resistance training [25] for unspecified constipation, were proposed.

For the genito-urinary system, postoperative rehabilitation that included mobilization and walking was proposed for unspecified constipation [22], while acupressure was recommended for opioid-induced constipation [34].

The methods of the assessments used to quantify treatment effects varied among studies, even for those exploring similar domains. These assessments aimed to show an improvement in symptoms related to constipation and overall quality of life. In particular, the effects of treatment on the severity of symptoms were very much studied. For instance, abdominal massage was evaluated using CAS [20,24], and electro-acupuncture was studied using the four-point Likert scale [30]. Acupuncture was analysed based on the Rome III diagnostic criteria, the Cleveland Clinic constipation score [26], and the constipation symptom score [29]. The effectiveness of electro-acupuncture was also gauged by the number of spontaneous bowel movements per week [31,32] and the Bristol stool form scale [30,31,32]. Auricular acupressure was evaluated with the stool-form score and CAS [28]. Finally, TENS was assessed using the bowel function index [19].

Quality of life was another frequently studied aspect. The EORTC QLQ-C-30 was used for evaluating the effects of massage [25], walking [22], and visceral osteopathic manipulation [23]. The EORTC QLQ-C-15 was used for TENS [27], the HRQoL for mobilization and walking [22], the MQOL-HK for massage [24], and the PAC-QOL for TENS, auricular acupressure, acupuncture and electro-acupuncture, abdominal massage and acupressure [19,26,28,29,31,32,33,34].

Anxiety and depression related to constipation were also explored. The HADS was used to assess the effects of massage and aerobic and resistance training [25]. The self rating anxiety scale [26] and the self-rating depression scale were used for electro-acupuncture stimulation [26].

Finally, patient satisfaction and expectations were evaluated for electro-acupuncture using the Patient’s Global Assessment of Treatment Effectiveness [31,32], and a satisfaction survey was used for mobilization and walking training [22].

While the current literature generally agrees on the beneficial effects and potential clinical applications of the strategies proposed for treating cancer-related constipation, it is necessary to contextualize the results with patient-specific clinical characteristics. These characteristics, such as fatigue, nausea, and level of autonomy, were less analysed in previous studies, but they could affect the adherence to treatment. Furthermore, the diverse causes of constipation may significantly influence therapy responses. As such, the outcomes should be stratified according to the specific drug causing the constipation or the determination that the constipation is related to low levels of motility.

Lastly, additional research is not only required to develop a common alternative therapeutic program, but also to establish a universally accepted clinical assessment methodology to achieve more homogenous results.

Another aspect warranting investigation is the role of cellular oxidative stress in cancer development [40]. Oxidative stress is associated with various diseases [41,42,43], including cancer [40]. On the contrary, regular, moderate and structured exercise, along with visceral therapy, appear to alleviate the negative effects caused by free radicals. These activities offer numerous health benefits, including reduced risk of all-cause mortality, skeletal muscle sarcopenia, chronic disease, constipation and premature death in elderly people [44,45,46]. Therefore, physiotherapy should be proposed as supportive therapy in patients with cancer [44]. Indeed, exercise and diet generally help to manage constipation [46]. However, physical activity can also induce oxidative stress, inflammation and muscle fatigue [44]. Consequently, micronutrients and natural compounds, commonly known as nutraceuticals, should be included in the regimen to prevent or mitigate exercise-induced oxidative stress and associated symptoms. For instance, supplementation with L-carnitine enhances body strength, sports endurance and exercise capacity, while delaying the onset of fatigue, a typical symptom related to cancer that hinders adherence to rehabilitation and physical exercise [47]. Therefore, specific strategies for alleviating constipation should be integrated into a broader project aimed at improving overall health in patients with cancer.

## 5. Limitations

Unfortunately, the limited number of articles and lack of homogeneous, quantitative data prevented the execution of a meta-analysis. Therefore, not all questions have been adequately addressed.

First, can rehabilitation, osteopathy and acupuncture improve constipation in patients affected by cancer? According to the available literature, they can. However, the determinations of the type of therapy and the ideal timing for each approach cannot be definitively and clearly explained.

Secondly, does the response to treatment depend on the pathophysiology of constipation (i.e., drug-induced constipation versus functional constipation)? Several articles included in this systematic review investigated the role of rehabilitation strategies, osteopathy and acupuncture in opioid-induced constipation. However, others did not specify the type of constipation under study.

Lastly, is there a variation in treatment response based on the location or stage (severity) of the primary tumour? More evidence is needed to answer this question.

Indeed, despite the total number of patients affected by cancer across all included studies, a population amounting to 6757, the heterogeneity of data regarding the proposed therapeutic strategies, in terms of types and timing, causes of constipation (primary or secondary to different drugs or related to low levels of motility), severity of symptoms associated with constipation, and the varied location and stages of tumours, complicates quantitative comparisons of the results. Specifically, the cause of constipation, which is not always investigated, can significantly influence treatment responses (i.e., constipation related to low levels of motility may respond better to physical activity).

While it doesn’t appear that the tumour site represents a significant variable, the proposed strategies should still consider this aspect to avoid biases related to symptoms associated with a specific tumour (i.e., gastrointestinal tumours could cause bowel dysfunctions, a confounding factor for the results).

Another significant challenge of the study is the high variability of clinical assessments. Although the investigated domains are similar (severity of symptoms associated with constipation, quality of life, anxiety, and depression mood in cancer patients), the lack of homogeneity in the scales used precludes a quantitative analysis that could better clarify the impact of the proposed strategies.

Furthermore, the lack of descriptions of patients’ clinical characteristics and the tumour stages are significant limitations of the study, as associated symptoms (such as nausea, fatigue, level of autonomy, presence of metastasis, etc.) could negatively influence the outcomes.

## 6. Conclusions

Constipation is a common problem among cancer patients, and a challenging one to prevent.

The use of abdominal massage/manipulation and personal care management could be recommended, provided that specific training is given to the patient or caregiver in order to manage constipation. Physical exercises could be included if the clinical conditions of cancer patients permit. Acupressure, acupuncture and TENS could be incorporated when administered by an expert operator.

Regrettably, the current literature does not enable a determination of which strategies work best, how much to recommend, what type of constipation responds better to the proposed treatments (functional or secondary), or how much the severity of symptoms, the tumour’s localization, and the stage influence the outcomes.

Although rehabilitation and alternative strategies may provide benefits for constipation in oncological patients, further studies are needed to overcome the limitations of the current literature. These studies should aim to establish the optimal protocols, their timing and lasting effects, and determine the influences of cancer stage, location and the cause of constipation on the results.

## Data Availability

Not applicable.
